# Determinant of repeat revascularization within 5 years of Percutaneous Coronary Intervention at a tertiary care hospital, Karachi: A matched case-control study

**DOI:** 10.1016/j.amsu.2022.103364

**Published:** 2022-02-11

**Authors:** Komal Valliani, Azmina Artani, Iqbal Azam, Javed Tai, M. Masood Kadir

**Affiliations:** a Aga Khan Development Network Digital Health Resource Centre, Aga Khan University, Karachi, Pakistan; b Department of Medicine, Aga Khan University, Karachi, Pakistan; c Department of Community Health Sciences, Aga Khan University, Karachi, Pakistan

**Keywords:** Repeat revascularization, Percutaneous coronary intervention, Coronary artery disease, AHA, American Heart Association, BMS, Bare Metal Stent, BMI, Body Mass Index, CI, Confidence Interval, CABG, Coronary Artery Bypass Grafting, CAD, Coronary Artery Disease, DBP, Diastolic Blood Pressure, DES, Drug Eluting Stent, HIMS, Hospital Information Management System, ISR, In-stent restenosis, IQR, Inter-quartiles range, JCI, Joint Commission International, MACE, Major Adverse Cardiac Events, MOR, Matched Odds Ratio, PCI, Percutaneous Coronary Intervention, SBP, Systolic Blood Pressure

## Abstract

**Objective:**

To determine factors associated with repeat revascularization among adults aged 25 years and above within 5 years of first Percutaneous Coronary Intervention (PCI) at a tertiary care hospital.

**Methods:**

A matched case-control study was conducted through a hospital records review. A total of 90 cases with repeat revascularization and 180 controls without repeat revascularization were included. Data was analyzed using Multiple Conditional Logistic Regression.

**Results:**

The mean age was similar in cases and controls (60.05 ± 10.01 vs 62.20 ± 10.43 years) and sex (male: 77.8% vs. 76.1%). History of being an ever-smoker (40% vs. 25%), overweight (36.3% vs. 30.6%), and poor glycemic control (23.3% vs. 12.2%) were more among the cases than controls. However, obesity (53.7% vs. 44.3%) and pre-diabetes (16.1% vs. 7.8%) were more in controls compared to cases.

Upon matching on the time of index PCI, the adjusted odds of ever smokers among patients with repeat revascularization was 2.47 times the odds of ever smokers among patients who did not undergo revascularization. Increasing stent diameter by 1 mm was found to reduce the risk of repeat revascularization by 51%.

**Conclusions:**

Smoking cessation and appropriate selection of stent diameter in patients undergoing revascularization can reduce the risk of repeat revascularization in the future.

## Highlights

• Study assessed determinants of repeat revascularization after initial PCI from a large tertiary care hospital in Karachi.
• Ever smokers undergoing index PCI have 2.47 times increased risk of repeat revascularization.
• 1 mm increase in stent diameter reduces 51% risk of repeat revascularization.
• Association between smoking and repeat revascularization may be a severe problem than estimated due to the reporting bias.
• More effective and well-tolerated strategies are needed for smoking cessation and sustain abstinence.

## 1 Introduction

Percutaneous Coronary Intervention (PCI) is a common procedure to revascularize coronary arteries. It is a nonsurgical invasive procedure that restores blood flow to the heart (revascularization). It opens arteries that are constricted by atherosclerotic plaque. Over time, advanced procedural techniques and adjunct pharmacological therapies have resulted in better outcomes post PCI, repeat revascularization remains a significant cause for readmission after initial revascularization. Repeat revascularization is defined as repeating the intervention which could be PCI or Coronary Artery Bypass Grafting (CABG) for restoring blood flow to the coronary arteries once a patient has been discharged after first or index PCI [1]. The requirement for repeat revascularization can differ depending on the risk and individual characteristics of a patient.

Repeat revascularization is often studied as an outcome or endpoint in Major Adverse Cardiac Events (MACE) after undergoing PCI or in comparative studies such as PCI vs. CABG [2]. Globally, few trials have been conducted which reported its incidence rate between 9 and 12% annually [[2], [3], [4], [5]]. A similar incidence rate was observed in the EVENT registry conducted in 55 centers of the United States, which reported a 12% incidence of repeat revascularization within a year. The 9% of repeat procedures were unplanned [5]. The studies concerning the risk of repeat revascularization, however, are limited indeed and risk factors identified are usually restricted to specific patient groups such as patients suffering from chronic kidney disease, diabetes, or other groups of diseases [[6], [7], [8], [9], [10]].

Coronary Artery Disease (CAD) affects South Asian descent to a greater extent due to markedly worse risk factor profile and more extensive disease [11]. It presents earlier in age and is associated with disease progression after index PCI and contributes to the high cardiovascular death rates in the region [12]. Studies conducted have reported the occurrence of hypertension, diabetes, hyperlipidemia, and a history of CAD to present even earliest than 40 years of age [[13], [14], [15]]. Also, recurrent myocardial ischemia was common within 12 months of index PCI [16]. A study conducted at a tertiary care hospital in Karachi identified that 34 out of 610 patients undergoing CABG had the previous stenting. Re-intervention was required due to aggressive disease progression and restenosis [17].

This study aimed to identify risk factors of repeat revascularization after undergoing index PCI including patient characteristics, comorbidities, smoking behavior, procedure related, and other such factors among adults aged 25 years and older visiting tertiary care hospital.

## 2 Methods

### 2.1 Study design

Matched case-control study design was employed to determine the association between repeat revascularization and its determinants in patients within 5 years of undergoing index PCI. The case was defined as a patient who had undergone repeat revascularization within 5 years of undergoing index PCI. Control was defined as a patient who had not undergone repeat revascularization within 5 years of undergoing index PCI.

### 2.2 Study setting

The retrospective study was carried out with the approval of the Ethical Review Committee of the tertiary care hospital where the study was carried out. A waiver of informed consent was granted as this was a retrospective study and all patients were discharged from the hospital. No personal identifiers were included in data collection, and records were anonymized to the statistician. To reduce the variability due to technique and its related factors, this site was selected as it follows the international guidelines and protocol including Joint Commission International (JCI) accreditation and the American Heart Association (AHA) which provide evidence-based Clinical Practice Guidelines [18]. These practices are standardized throughout the hospital that is ensured by internal and external quality audits. This article has been submitted in line with the STROCSS guidelines [19]. and has been registered with the Research Registry with a (UID: NCT05189249). https://clinicaltrials.gov/ct2/show/NCT05189249.

### 2.3 Participants

A minimum of 120 cases with matched 240 controls were required in 1:2 case to control ratio. This sample size was essential to achieve the power of 80% for an anticipated matched odds ratio of 2 with the hypothesized correlation of 0.2 using a two-sided hypothesis test with a significance level of 0.05.

#### 2.3.1 Eligibility criteria for cases

Inclusion criteria for cases included patients aged 25 years and older and who had undergone repeat revascularization within 5 years of undergoing index PCI (from 2011 to 2017). However, patients were excluded from being a case if suffering from any hypercoagulable disorder, revascularization procedure was performed outside the study setting, had staged PCI within 3 weeks or planned PCI within 6 months of Index PCI. The staged procedure was defined as the planned PCI once a patient has been discharged after the index procedure [20].

#### 2.3.2 Eligibility criteria for controls

Inclusion criteria for controls included patients aged 25 years and older and who had undergone PCI once from the year 2011–2017. However, patients were excluded from the control group if they had any hypercoagulable disorder.

The non-probability consecutive sampling strategy was used to identify patients from the provided list of medical records. Patients were recruited in the study if they fulfilled the eligibility criteria for being a case or control and were selected until the required sample was achieved. Previous exposures were subsequently explored for each patient. For controls, the tool assessed the exposure status after index angioplasty while for cases, the tool assessed the exposure status post-angioplasty and before undergoing repeat revascularization.

#### 2.3.3 Matching variable

The time of index PCI could be a potential confounder in this study. The reason behind undergoing repeat procedures within a year of index PCI could be different than undergoing the procedure after a few years of index PCI. This was handled by matching the time of undergoing initial PCI in both study groups that is the year of undergoing index PCI. For instance, a case that had undergone index PCI in the year 2012 was matched with two controls who had index PCI in 2012 i-e 1:2 case to control ratio.

### 2.4 Outcome and study variables

The outcome of our study was the Repeat revascularization status. There are different types of repeat revascularization according to the site and lesion to which intervention has been provided. Any type of repeat revascularization was considered and enrolled as a case in this study [5].

Covariates included in the study were divided into patient characteristics (gender, age, health coverage, Body Mass Index (BMI), smoking status), comorbidity status (Hypertension, Diabetes Mellitus, Hyperlipidaemia, valvular disease), clinical characteristics (Creatinine level, Systolic Blood Pressure (SBP), Diastolic Blood Pressure (DBP), HbA1c, Cholesterol level, other technical factors related to the index PCI) and medication status (Beta-blocker, ACE Inhibitor, Statin).

For BMI, well-established biological cutoffs were available. The Asian cutoff ranges a BMI lower than 18.5 kg/m^2^ suggests the person is underweight, a BMI from 18.5 up to 23 kg/m^2^ indicates the normal weight, from 23 up to 27.5 kg/m^2^ indicates the person is overweight, and from 27.5 kg/m^2^ upwards suggests the person is obese [21,22]. Moreover, the AHA defined categories for left ventricular ejection fraction [23] and the biological cutoff was used for average HbA1c.

### 2.5 Statistical methods

All the analysis was carried out using STATA software (version 13.0). For normally distributed quantitative variables, mean and standard deviation are reported whereas, median and inter-quartiles range (IQR) are stated for variables deviating from normality. Frequencies and proportions are reported for qualitative variables.

Variables were regressed with the Repeat revascularization status using simple Conditional Logistic Regression. Univariate analysis was conducted by computing Crude Matched Odds Ratio (MOR) and their 95% Confidence Interval (CI) to compare cases and controls for different factors. The cutoff of p-value <0.25 was considered as significant at the univariate level to be eligible for multivariable analysis. Multicollinearity was assessed between covariates at the cutoff equal to or more than 0.8.

All independent variables were regressed with the outcome through Multiple Conditional Logistic Regression by using the stepwise method. With each extension of the model, the likelihood ratio test was used to decide the inclusion of further variables which are considerably improving the fit of the model. Matched Odds Ratios and 95% Confidence Interval (CI) were reported for statistically significant variables in the final model. A p-value of less than or equal to 0.05 was considered significant.

## 3 Results

### 3.1 Descriptive data

A total of 1055 patient record files were reviewed, from which 90 cases and 180 matched controls were identified meeting the eligibility criteria, and were included in the study. The mean age of the participants (60.05 ± 10.01 vs 62.20 ± 10.43 years) and the proportion of males (77.8% vs. 76.1%) was found similar in cases and controls (Table 1). History of being ever smokers (40% vs. 25%), and overweight (36.3% vs. 30.6%) was more common among cases than controls. However, non-smokers (66.7% vs. 44.4%), self-payers of healthcare (93.3% vs. 78.9%), and obesity (53.7% vs. 44.3%) were more common in controls compared to cases.

**Table 1:** Characteristics of the study participants according to their repeat revascularization status.

Variable	Repeat Revascularization Status		
	Cases (n = 90)	Controls (n = 180)	P-value
	n (%)	n (%)	
**Patient characteristics**			
**Male gender**	70 (77.8)	137 (76.1)	0.76
**Age (Years)**^a^	60.05 (10.01)	62.20 (10.43)	0.01
**Health coverage status (self-payers)**	71 (78.9)	168 (93.3)	0.01
**Weight (in kg)**^a^	70.66 (13.26)	73.91 (14.35)	0.09
**Body Mass Index**			
Under or Normal	17 (19.3)	25 (15.6)	0.45
Over weight	32 (36.3)	49 (30.6)	
Obese	39 (44.3)	86 (53.7)	
**Smoking status**			
Non-smoker	40 (44.4)	120 (66.7)	0.01
Ever smoker	36 (40.0)	45 (25.0)	
Not reported	14 (15.6)	15 (8.3)	
**Smokeless Tobacco Status**			
Never user	69 (76.7)	147 (81.7)	0.19
Ever user	7 (7.8)	18 (10.0)	
Not reported	14 (15.6)	15 (8.3)	
**Alcohol user**			
Never user	71 (78.9)	160 (88.9)	0.09
Ever user	5 (5.6)	5 (2.8)	
Not reported	14 (15.6)	15 (8.3)	

a Mean (Standard Deviation).

Comorbidity status was approximately similar in both groups. Among clinical characteristics, around half of the participants in cases and controls had normal HbA1c or good glycemic control (Table 2). On the other hand, more cases were found with poor glycemic control with HbA1c greater than 8.5% than in controls (23.3% vs. 12.2%). In contrast, controls were found to have a higher number of patients with pre-diabetes (16.1% vs. 7.8%) and cholesterol level (136.87 ± 31.58 vs. 130.90 ± 30.17 mg/dl) as compared to cases.

**Table 2:** Clinical characteristics of study participants according to their repeat revascularization status.

Variables	Repeat Revascularization Status		
	Cases (n = 90)	Controls (n = 180)	P-value
	n (%)	n (%)	
**Comorbidity status**			
**Valvular disease**			
No disease	36 (32.2)	61 (33.9)	0.13
Mild	26 (28.9)	50 (27.8)	
Moderate	11 (12.2)	23 (12.8)	
Severe	0 (0.0)	5 (2.8)	
Not Available	15 (16.7)	41 (22.8)	
**Clinical Characteristics**			
**SBP (in mmHg)**^a^	123.50 (18.67)	128.62 (17.81)	0.02
**DBP (in mmHg)**^a^	69.73 (8.15)	69.73 (8.15)	0.33
**HbA1c**			
Normal <5.7%	45 (50.0)	99 (55.0)	0.06
Pre-diabetic 5.7–6.5%	7 (7.8)	29 (16.1)	
6.5–7.5%	13 (14.4)	19 (10.6)	
7.5–8.5%	4 (4.4)	11 (6.1)	
>8.5%	21 (23.3)	22 (12.2)	
**LDL (in mg/dl)**^b^	68.25 (42–123.66)	71.25 (36–138.4)	0.92
**HDL (in mg/dl)**^b^	35 (24–52)	38 (26–59)	0.35
**Triglyceride (in mg/dl)**^b^	135 (75–252)	118 (72.33–244.4)	0.06
**Number of Diseased vessels**			
I	41 (45.6)	100 (55.6)	0.29
II	35 (38.9)	56 (31.1)	
III	14 (15.6)	24 (13.3)	
**Number of stents**			
Single	58 (64.4)	107 (59.4)	0.43
Double	24 (26.7)	48 (26.7)	
Multi	8 (8.9)	25 (13.9)	
**Type of stents**			
Drug Eluting Stent (DES)	69 (76.7)	129 (71.7)	0.41
Bare Metal Stent (BMS)	16 (17.8)	44 (24.4)	
Both (DES & BMS)	5 (5.6)	7 (3.9)	
**Stent Diameter (in mm)**^b^	2.82 (0.37)	2.91 (0.42)	0.03
**Stent Length (in mm)**^b^	22 (15–38)	21.5 (12–40)	0.11
**Ejection Fraction (Valvular Heart Disease)**			
Normal	6 (17.8)	8 (4.4)	0.45
Mild	35 (38.9)	59 (32.8)	
Moderate	14 (15.6)	36 (20.0)	
Severe	19 (21.1)	32 (17.8)	
Echo not recommended	16 (17.8)	45 (25.0)	

a Mean (Standard Deviation).

b Median (Inter-quartiles range).

Medication status post-index PCI was also found similar among cases and controls. It included glycoprotein IIb/IIIa Inhibitors (51.1% vs.54.4%), ACE inhibitor (47.8% vs.53.3%), angiotensin II receptor blocker (ARBs) (14.4% vs.12.2%), beta-blocker (88.9% vs 93.9%), statin (95.6% vs 98.3%) and anticoagulants (8.9% vs. 5%) respectively. All of the patients were prescribed dual antiplatelet at the time of discharge.

### 3.2 Main results

To perform conditional logistic regression, univariate analysis was performed by regressing independent variables with the outcome. Age was found to be significant with the crude MOR 0.98 (95% CI l 0.95–1.01). Other significant variables include health coverage, weight, smoking status, smokeless tobacco, alcohol user, valvular disease, SBP, HbA1c, triglyceride, number of diseased vessels, stent diameter, beta-blocker, statin and anti-coagulant. Statistically significant variables at this stage were selected for the multivariable model and those variables were entered first which had the least p-value.

In the final multivariable model, smoking status, health coverage status and stent diameter were found statistically significant by keeping all other variables constant (Table 3). Upon matching on the time of index PCI, the adjusted odds of current smoker among repeat revascularized patients was 2.47 times than the odds of current smoker in a patient who was revascularized once.

**Table 3:** Multiple Conditional Logistic analysis showing factors associated with repeat revascularization.

Variable	Category	Adjusted Matched Odds Ratio	95% Confidence Interval	p-value
Smoking Status	Non-smoker	**1**		
	Ever smoker	2.47	1.34–4.55	0.01
	Not reported	2.43	1.01–5.84	0.05
Health coverage status	Self-pay	**1**		
	Third party payer	3.27	1.51–7.08	0.01
Stent diameter		0.49	0.25–0.99	0.05

## 4 Discussion

Repeat revascularization is one of the areas in which very few studies have been conducted in South Asia. This study is one of the initial studies which aimed to identify the key determinants that are associated with repeat revascularization in CAD patients after undergoing initial PCI from a large tertiary care hospital. Our study reports smoking status, health coverage status and stent diameter as the factors affecting the outcome of index PCI in our study population and were related to the repeat revascularization.

Similar to the study findings, other studies have reported ever smokers undergoing PCI have an increased risk of repeat revascularization than those who have either stopped smoking or have never smoked [24]. Smoking is a modifiable risk factor and approximately 10–30% of the patients with known CAD continue their smoking habit; however, not all current smokers remain smokers after a coronary event or an intervention. Patients have benefited from smoking cessation counseling offered post-procedure and have reduced the proportion of current smokers to half [24]. Furthermore, a study conducted in the United States reported that smoking cessation counseling for current smokers at the time of index PCI reduced the first thirty-days mortality by 23% and over seventeen years by 8%. This resulted in an average gain of 0.13–0.58 years of life which is the largest ever reported for old age smokers [25]. Hence, smoking cessation counseling after index PCI should be incorporated as a mandatory component in discharge teaching as it can help to sensitize patients towards the issue and assist them in reducing or quitting smoking. This will decrease the chance of future repeat revascularization.

Health coverage is an important aspect of the healthcare system. Our study showed that the patients with health coverage presented more frequently for repeat revascularization than those who were self-payer; hence, were associated with a higher odds ratio. Insured patients are less worried about their healthcare cost coverage, they might have regular follow-ups and adherence to the physician's order [26]. In contrast, financial concerns might discourage people from coming to a cardiac facility. This relationship has not been studied extensively in our settings which prompts for further search.

Also, stent diameter has emerged as a significant factor in this study. Inappropriate selection of stent diameter could be a great threat to repeat revascularization. Increasing stent diameter by 1 mm was found to reduce the risk of repeat revascularization by 51%. A similar finding was reported in the BASKET-PROVE trial that was conducted in four countries. It stated that the risk of In-stent restenosis (ISR) decreased by 76% with an increase in stent diameter by 1 mm [27]. Besides, another study conducted on a large cohort of ethnically diverse patients undergoing PCI with DES reported smaller stent diameter to be associated with increased MACE, leading to higher rates of repeat revascularization [28].

Since, CAD accounts for a significant burden of morbidity, mortality and health expenditures in low-middle income countries; public health personal should focus on developing relevant risk assessment tools, cost-effective prevention and therapeutic strategies [29]. As smoking is associated with poor outcomes post-angioplasty, this information is significant for patients, physicians and the general population. It is important to inquire about smoking status at each clinical encounter to counsel patients for quitting their habit [25]. Although these patients remain at risk of relapse, additional efforts are required to develop more effective and well-tolerated strategies to assist cessation and sustain abstinence from it. Health coverage and its association are less studied in repeat revascularization studies. Moreover, there are only limited data available to examine the effect of stent diameter in our routine clinical practice. These require further investigation to study its role in the occurrence of repeat revascularization in our population.

It also suggested that the future large-scale multicenter prospective studies should be conducted to evaluate the role of exercise, medication adherence, lifestyle modification and the role of biochemical milieu with repeat revascularization in our setting which is likely to give further major contributions.

### 4.1 Strengths and limitations

In our population, research pertinent to repeat revascularization has been very limited. This study is an initial effort to explore the factors that may be related to the outcome of repeat revascularization following an initial PCI and hence would set the stage for future studies. In addition, the study was conducted in a JCI accredited tertiary care hospital and the availability of a well-maintained systematic record system at the hospital helped us to extract study data and clinical characteristics of patients more comprehensively.

The study has certain limitations. As the study was conducted at a single center, the findings of the study are limited in generalizability to the CAD patients in the community. Being a case-control study, it was at potential risk of selection bias. As hospital-based cases represent a more severe form of the disease, controls were also identified from the same list from which cases were selected in an attempt to minimize this bias. Furthermore, potential misclassification bias may exist because cases might have opted for repeat revascularization outside of the study site. The lack of an integrated healthcare system in the city has made us rely entirely on the Hospital Information Management System (HIMS) data. However, information was thoroughly reviewed that was available in the patient's file.

It is also a possibility that a reporting bias may have occurred to certain variables that are socially less desirable and are associated with stigmas, such as alcohol consumption and smoking. This could have diluted the difference among cases and controls, and the impact reported might be an underestimation of association with repeat revascularization. Nevertheless, the study suggests an association between smoking and repeat revascularization which may be a more severe problem than estimated in the study [25].

## 5 Conclusion

Risk factors play a vital role in the disease progression after index PCI and in the occurrence of repeat revascularization. Smoking and smaller stent diameter were noted to be associated with higher rates of repeat revascularization in our study population. These findings highlight the significance of targeted strategies aiming at patients undergoing index coronary intervention. Appropriate selection of stent diameter is crucial in reducing the risk of repeat revascularization. Incorporation of smoking cessation counseling in discharge teaching and inquiry of smoking status at each clinical encounter is important to provide more effective and well-tolerated strategies to assist cessation and sustain abstinence. Future prospective multicenter studies are required to assess role of other related risk factors to help improve the outcomes of patients after PCI.

## Ethical approval

The ethics committee of the Aga Khan University approved this study (5004-CHS-ERC-17).

## Sources of funding

The authors received no specific funding for this work.

## Author contributions

KV: Conceptualization, Methodology, Investigation, Project administration, Formal analysis, Data curation, Writing - Original Draft.

AA: Project administration, Formal analysis, Writing - Review & Editing.

IA: Formal analysis, Writing - Review & Editing.

JT: Clinical Expertise, Conceptualization, Writing - Review & Editing.

MK: Supervision, Conceptualization, Methodology, Writing - Review & Editing.

## Trial registry number

1. Name of the registry: ClinicalTrials.gov.

2. Unique Identifying number or registration ID: NCT05189249.

3. Hyperlink to your specific registration (must be publicly accessible and will be checked): https://clinicaltrials.gov/ct2/show/NCT05189249.

## Guarantor

Ms. Komal Valliani.

## Consent

Not applicable.

## Data sharing statement

The data that support the findings of this study are available from the corresponding author upon reasonable request.

## Informed consent

Not applicable.

## Provenance and peer review

Not commissioned, externally peer-reviewed.

## Declaration of competing interest

The authors declare that they have no conflicts of interest.
